# Longitudinal Patterns and Predictors of Racial Disparities of HIV Retention in Care: A Statewide Cohort Analysis

**DOI:** 10.1007/s10461-025-04813-9

**Published:** 2025-07-16

**Authors:** Fanghui Shi, Chen Zhang, Jiajia Zhang, Bankole Olatosi, Sharon Weissman, Xiaoming Li, Xueying Yang

**Affiliations:** 1 Arnold School of Public Health, South Carolina SmartState Center for Healthcare Quality, University of South Carolina, 915 Greene Street, Columbia, SC 29208 USA; 2 Department of Health Promotion, Education and Behavior, Arnold School of Public Health, University of South Carolina, Columbia, SC 29208 USA; 3 Department of Epidemiology and Biostatistics, Arnold School of Public Health, University of South Carolina, Columbia, SC 29208 USA; 4 Department of Health Services Policy and Management, Arnold School of Public Health, University of South Carolina, Columbia, SC 29208 USA; 5 Department of Internal Medicine, School of Medicine, University of South Carolina, Columbia, SC 29208 USA

**Keywords:** HIV, Retention in care, Racial disparity, South Carolina

## Abstract

Racial disparities have historically existed regarding HIV care outcomes, including retention in care (RIC). However, data regarding the longitudinal patterns and predictors of such disparities among different racial groups and various geolocations are limited. This study aims to explore the contribution of contextual features (e.g., socioeconomic and structural environmental factors) to the changes in RIC outcome disparity at the county level. All adult people with HIV who received a HIV diagnosis in South Carolina (SC) between 2013 and 2020 were included in this study, and their de-identified CD4 counts and viral load information were derived from the SC statewide HIV registry system. All contextual factors were extracted from multiple publicly available datasets, such as the American Community Survey. To provide a comprehensive view, we used four indices to measure racial disparities in the county-level percentage of RIC, i.e., Black to White ratio (BWR), Index of Disparity (ID), Weighted Index of Disparity (Weighted ID), and Gini coefficient. Linear mixed-effect models were used to estimate the relationship between various contextual factors and these disparity indexes, except BWR. The temporal trend of the four indices suggested no significant increase or decrease in racial disparities in RIC across 46 counties in SC from 2013 to 2020. Counties with more primary care providers and higher social capital in collective efficacy showed lower racial disparities in RIC when ID and Gini coefficient were used as outcomes. For ID only, higher racial disparities in RIC were observed in counties with lower isolation index (β=-1.92, 95%CI: -3.31~-0.53). For weighted ID only, no county-level contextual features explored were significantly associated with racial disparities in RIC. Racial disparities in RIC persist without major changes in SC despite the national implementation of the “Ending the HIV Epidemic” initiative. Efforts to address racial disparities should continue; specifically, efforts that focus on social and structural factors should be developed and implemented for populations that have poor HIV outcomes in the US.

**Supplementary Information:**

The online version contains supplementary material available at 10.1007/s10461-025-04813-9.

## Introduction

Retention in care (RIC) is a critical step in the HIV care continuum, essential for improving health outcomes among people with HIV (PWH) and reducing HIV transmission [1]. PWH who receive consistent medical care are more likely to adhere to antiretroviral therapy, less likely to progress to AIDS, and exhibit higher survival rates compared to those without regular care [2, 3]. Furthermore, RIC can facilitate the suppression of HIV viral loads to undetectable levels, which is critical in preventing further transmission [4]. Despite these benefits, only 54% of people diagnosed with HIV in the United States (US) and alive at the end of 2022 were retained in care, with notable racial/ethnic disparities persisting [5–7]. Specifically, in 2022, the proportion of Black/African American PWH (52%) who are retained in care is lower than that among non-Hispanic White PWH (55%) [5, 7].

Racial disparities in RIC are prevalent among PWH [8]. A systematic review of disparities in RIC among adults living with HIV/AIDS summarized that non-Hispanic Blacks/African Americans exhibited increased risks of poor retention in care compared to non-Hispanic Whites, even after adjusting for individual (e.g., age, gender, and HIV transmission mode) [7] and neighborhood (e.g., transportation barrier and residential segregation) factors [8]. Additionally, the degree of racial disparities in RIC varied by geographic location, with the southern US showing more pronounced disparities than other regions [9]. Given the significant HIV burden and low RIC rate in the Southern US, there is an ongoing need for enhanced efforts to understand and address the factors contributing to racial/ethnic disparities in HIV RIC in this area, such as South Carolina (SC).

While numerous studies have examined the social and structural determinants of RIC among PWH [10, 11], limited research focuses specifically on the structural predictors of racial disparities in RIC. Understanding racial disparities in RIC needs to be framed within the context of social and structural factors, such as racial residential segregation, which shape PWH’s options and opportunities for social relationships, healthcare access, housing, and physical and mental health. Systemic barriers to RIC have been well documented in past research and include factors such as a lack of easily available housing and high-quality services, residing in geographic areas with poor socioeconomic conditions, inadequate transportation infrastructures that hinder access to care, and insufficient availability of comprehensive HIV care [12]. These barriers may disproportionately affect Black/African American PWH due to historical systemic racism, leading to increased racial disparities in HIV care outcomes [13–15]. However, it remains unclear whether these barriers contribute to a widening disparity in RIC between Black and White individuals, and which specific barriers have the greatest impact on these racial disparities.

Various measures have been used to quantify racial disparities in disease burden, such as rate ratio, index of disparity (ID), weighted ID, and Gini coefficient [16, 17]. The rate ratio compares the outcome rate of one racial group to a reference group, while the ID is an absolute summary measure that captures the average percentage difference between the outcome rates of each racial group and a reference group. Based on ID, the weighted ID incorporates the population size of each racial group to ensure that larger groups have a greater influence on the index. The Gini coefficient measures the extent to which the distribution of health outcomes among racial groups deviates from perfect equality. These disparity measures could be useful and informative in summarizing the burden of disparity at a time point, analyzing the temporal trend, and informing the target group of HIV prevention. However, there is potential for inconsistencies between different disparity measures because they differ in their data requirement, calculation method, and some other factors [16, 17]. For example, the 2011 Sexually Transmitted Disease Surveillance report from the Centers for Disease Control and Prevention presented 3 disparity measures, including ID, weighted ID, and the Gini coefficient, to illustrate racial disparities in gonorrhea rates between 2007 and 2010 [17]. In this report, an increase in disparity was indicated by ID, but a decrease in disparity was indicated by weighted ID and Gini coefficient. The selection of which disparity measures to choose requires subjective judgements by the user about what is just, fair, and socially acceptable [16, 18–21]. Experts in the field of quantifying disparities have suggested using multiple measures instead of relying on any single measure to provide a comprehensive view of racial disparities in disease burden [22]. Using multiple indices helps capture different aspects of disparities, guiding more effective and equitable public health interventions [22]. 

The present study sought to leverage integrated statewide electronic health records (EHRs) data and publicly available datasets to (1) use quantified measurements of racial disparities in RIC and explore their spatiotemporal variations across 46 counties in SC from 2013 to 2020 and (2) investigate social and structural factors associated with these disparities at the county level. The findings from this study can provide valuable insights to inform community- or structural-level interventions designed to reduce racial disparities in RIC across SC.

## Methods

### Participants and Data Sources

The current study is a statewide population-based retrospective cohort study conducted at the county level. All adult (≥ 18 years old) PWH who received a HIV diagnosis in SC across 46 counties from 2013 to 2020 were included in analyses. Their CD4/viral load test records were extracted from the SC Department of Public Health (DPH) Enhanced HIV/AIDS Reporting System (eHARS) [23], which is a statewide surveillance system developed by CDC to track population-based HIV/AIDS data. All county-level variables that measure the socio-environmental characteristics of the PWH were retrieved from publicly available survey/data sources, such as the American Community Survey (ACS), County Health Rankings & Roadmaps, and Area Health Resources File (AHRF). The Federal Information Processing Standards (FIPS) code, a numeric code unique to each county, was used to link aggregated county-level indicators through varied data sources. The University of South Carolina institutional review boards and relevant SC state agencies approved the study protocol (IRB #: Pro00121718).

### Racial Disparities in County-Level RIC

Individual-level variables in the SC HIV registry system between 2013 and 2020 were used to calculate the aggregated county-level retention in care (RIC) percentage. According to the CDC definition, individual-level RIC status was identified yearly and considered “retained in care” in a calendar year if two or more CD4/viral load tests were performed at least three months apart during that year [24]. Then, individual-level RIC status was aggregated to the county level, and the county-level RIC rate was measured as the percentage of PWH who were considered “retained in care” in each county during one calendar year. To capture different disparities, we used four commonly utilized relative measurements of racial disparities in county-level RIC, including the black-to-white rate ratio (BWR), ID, weighted ID, and Gini coefficient.

We calculated the BWR as the percentage of RIC among Black PWH divided by the percentage of RIC among White PWH in each county. A BWR greater than 1 indicates the RIC rate is higher for Black individuals compared to White individuals, highlighting a specific disparity between these two groups. The ID reflects the average of the absolute differences between RIC rates in different racial groups we examined (Black PWH and White PWH) and the RIC rate for the overall population. We calculated ID as follows:

$$\:ID=\:100*\left(\frac{{\sum\:}_{i=1}^{2}\left|{Rate}_{i}-{Rate}_{overall}\right|}{2}\right)/{Rate}_{overall}$$

where i indicates the racial group, Rate is the RIC rate among PWH in the given group, and Rate_overall_ is the RIC rate across Black and White PWH combined. Higher values of ID indicate greater variability and overall disparity.

We calculated weighted ID in an analogous manner, except that we calculated a population-weighted average of the absolute differences in RIC rates between each group and the RIC rate for the overall population, as follows:

$$ \begin{gathered} \:Weighted\:ID = \hfill \\ \:100*\left( {\frac{{\sum \: _{{i = 1}}^{2} \left| {Rate_{i} - Rate_{{overall}} } \right|*Population_{i} }}{{Population_{{overall}} }}} \right)/Rate_{{overall}} \hfill \\ \end{gathered} $$

where Population_i_ is the population size for group i and Population_overall_ is the population size of combined Black and White groups. Weighted ID gives more importance to disparities affecting larger groups, and higher values indicate greater disparities.

The Gini coefficient was adapted from a statistical measure of economic inequality in a population and was applied to sexually transmitted disease prevention-related research [25]. To calculate Gini coefficient, we ranked the Black and White groups 1 and 2 according to their RIC rates (i = 1 and i = 2 denote group with lower and higher rates, respectively). We calculated the Gini coefficient as:

$$\:G=1-\:{\sum\:}_{i=1}^{i=2}({Y}_{i}+\:{Y}_{i-1})({X}_{i}-\:{X}_{i-1})$$

where *Y*_*i*_ and *X*_*i*_ are the cumulative percentage of RIC and the cumulative percentage of the number of PWH, respectively, and *X*_*0*_ and *Y*_*0*_ are both 0. The Gini coefficient summarized overall inequality across racial groups, and higher values suggest a more unequal distribution of RIC.

The selection between the index of disparity (ID) and weighted ID depends on one’s choice of whether to treat each racial/ethnic group equally, regardless of population size. Both weighted ID and Gini coefficient account for the population size of the racial/ethnic groups, whereas the Gini coefficient reflects the overall spread of health disparities without focusing on specific groups, and weighted ID gives more weight to groups with greater population representation. Thus, the selection between weighted ID and Gini coefficients depends on whether to emphasize disparities affecting larger groups. Additionally, compared to ID, weighted ID, and Gini coefficient, the rate ratio directly measures how many times higher (or lower) one group’s rate is compared to the other, instead of reflecting the overall disparity between two groups.

### County-Level Characteristics

Based on the social determinants of health conceptual framework [26], a total of 24 county-level characteristics were retrieved from publicly available datasets (e.g., American Community Survey, County Health Rankings, and U.S. Department of Health and Human Services), and they were categorized into intro five groups, including (1) racial residential segregation: spatial proximity, delta, Black/White dissimilarity index, and isolation index; (2) social capital indices: collective efficacy, institutional health, community health, and family unity; (3) social vulnerability indices (SVIs): socioeconomic status, minority status and language, household characteristics and disability, and housing type and transportation; (4) health care resources and health behavior: the number of primary care providers, Ryan Whtie HIV centers, and mental health centers, and the percentages of smoking, drinking, and disability; (5) other characteristics: male%, vacant houses%, unemployed%, Gini index for income inequality, uninsured%, and religious adherence%. The detailed definitions and calculations are listed in supplemental Table 1. Additionally, 46 counties were categorized into four public health regions defined by SC DPH, including Upstate, Midlands, Pee Dee, and Lowcountry.

### Statistical Analyses

First, we used the 25th percentile, median, 75th percentile, and interquartile range (IQR) to summarize county-level characteristics in 2020. Second, we employed line plots with linear regression analyses to demonstrate the temporal trend of four racial disparities in RIC indices from 2013 to 2020. Third, we generated maps to depict the spatiotemporal variations of the ID and Gini coefficient in SC in 2014, 2016, 2018, and 2020. Finally, we applied linear mixed-effects models with forward selection to investigate the relationship between county-level characteristics and three racial disparities in RIC measurements, including ID, weighted ID, and Gini. The random effect accounted for repeated measures in the same county. The Black to White ratio was not included as an outcome in the analysis because the Black to White ratio does not reflect racial disparities in a linear manner, and linear mixed effects models could oversimplify the complex non-linear relationship between county-level characteristics and racial disparities. All analyses were conducted using R version 4.1.2, and the statistical significance level was set at a p-value less than 0.05.

## Results

### Spatiotemporal Variation of County-Level Disparities in RIC

**Fig. 1:**
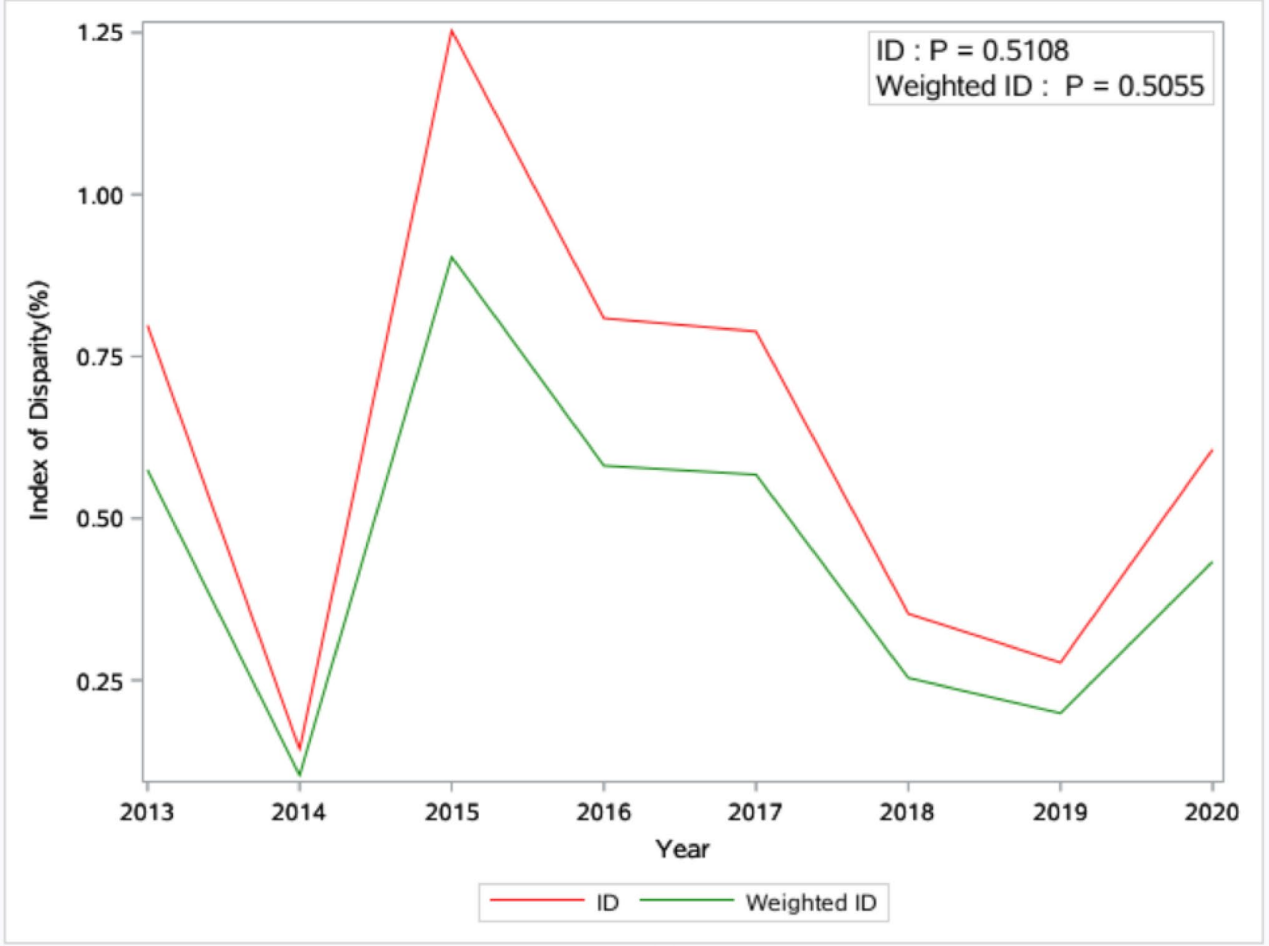
Temporal trend of Index of Disparity (ID) and Weighted version of the Index of Disparity (Weighted ID) from 2013 to 2020 in South Carolina

**Fig. 2:**
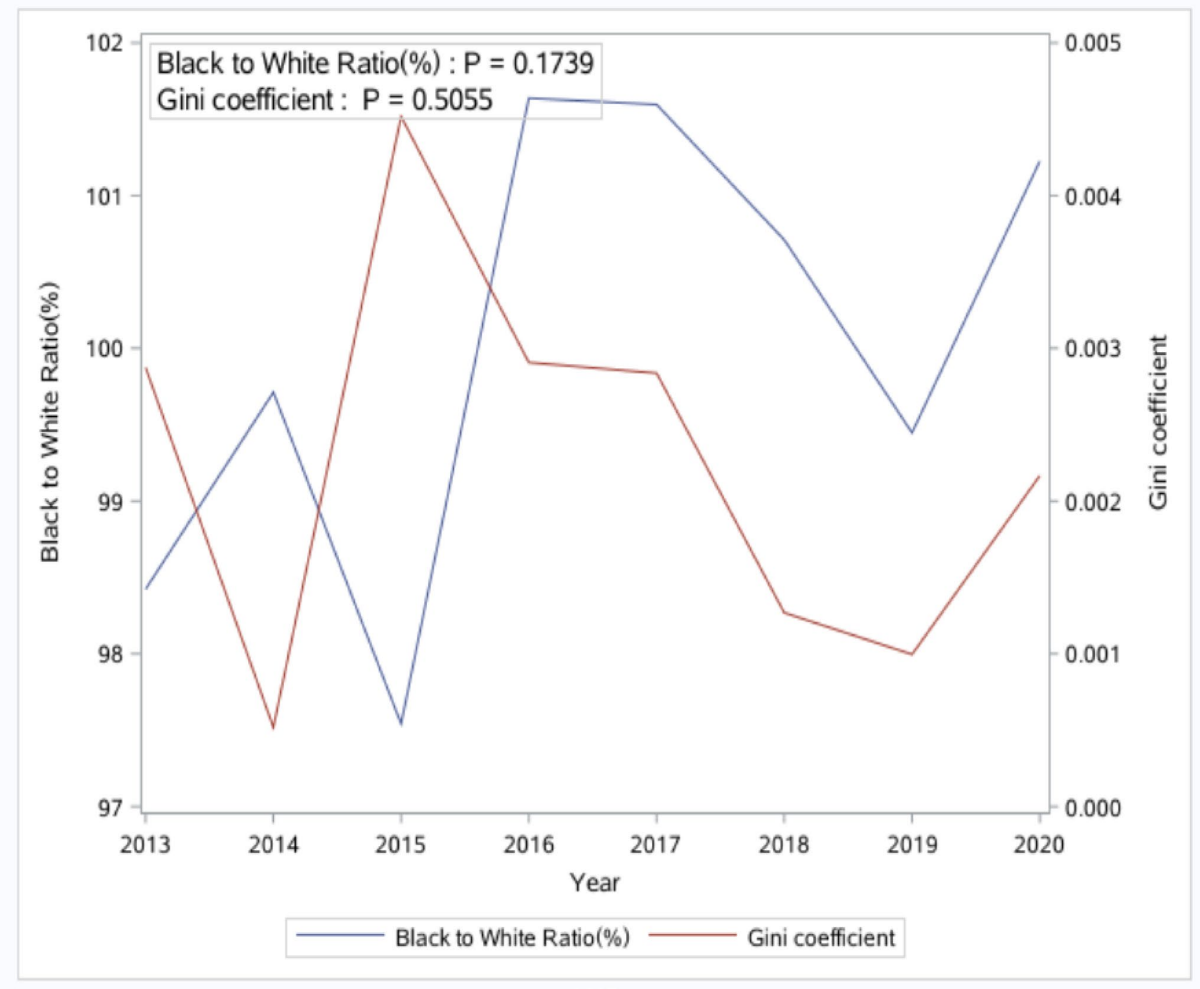
Temporal trend of Black to White ratio and Gini Coefficient of retention in care from 2013 to 2020 in South Carolina

From 2013 to 2020, a total of 17,591 adult PWH were included in the analyses, and most of them were male (72.2%), non-Hispanic Black (75.9%), and with the HIV transmission mode being men who have sex with men (45.2%). (Supplemental Table 2) The distribution of PWH across the 46 counties varied considerably, with a median of 100 PWH per county (range: 14 to 2,344; interquartile range: 166). There was no consistent increase or decrease in racial disparities in RIC over the years. (Figures 1 and 2).

**Fig. 3:**
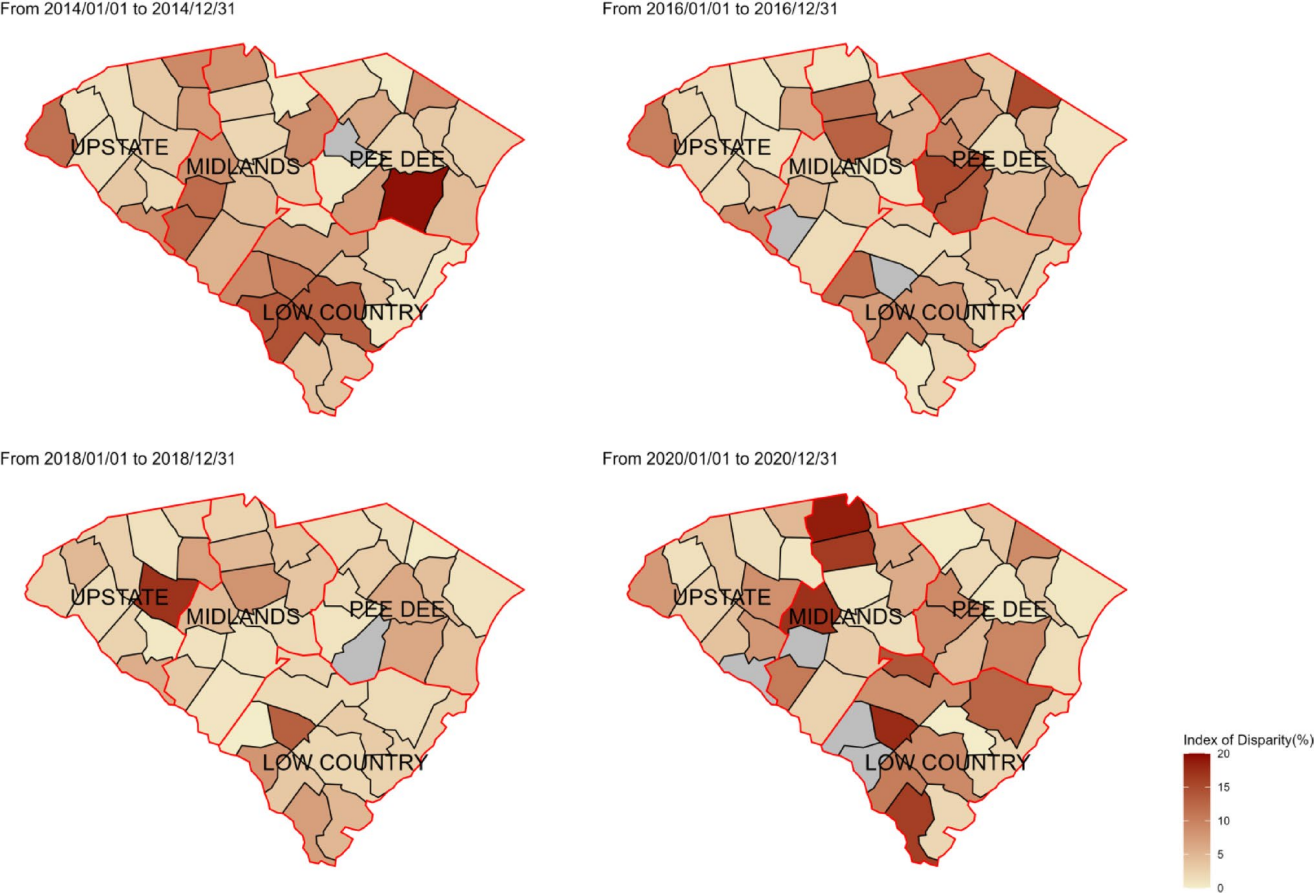
Geographic mapping for the index of disparity in South Carolina in 2014, 2016, 2018, and 2020

**Fig. 4:**
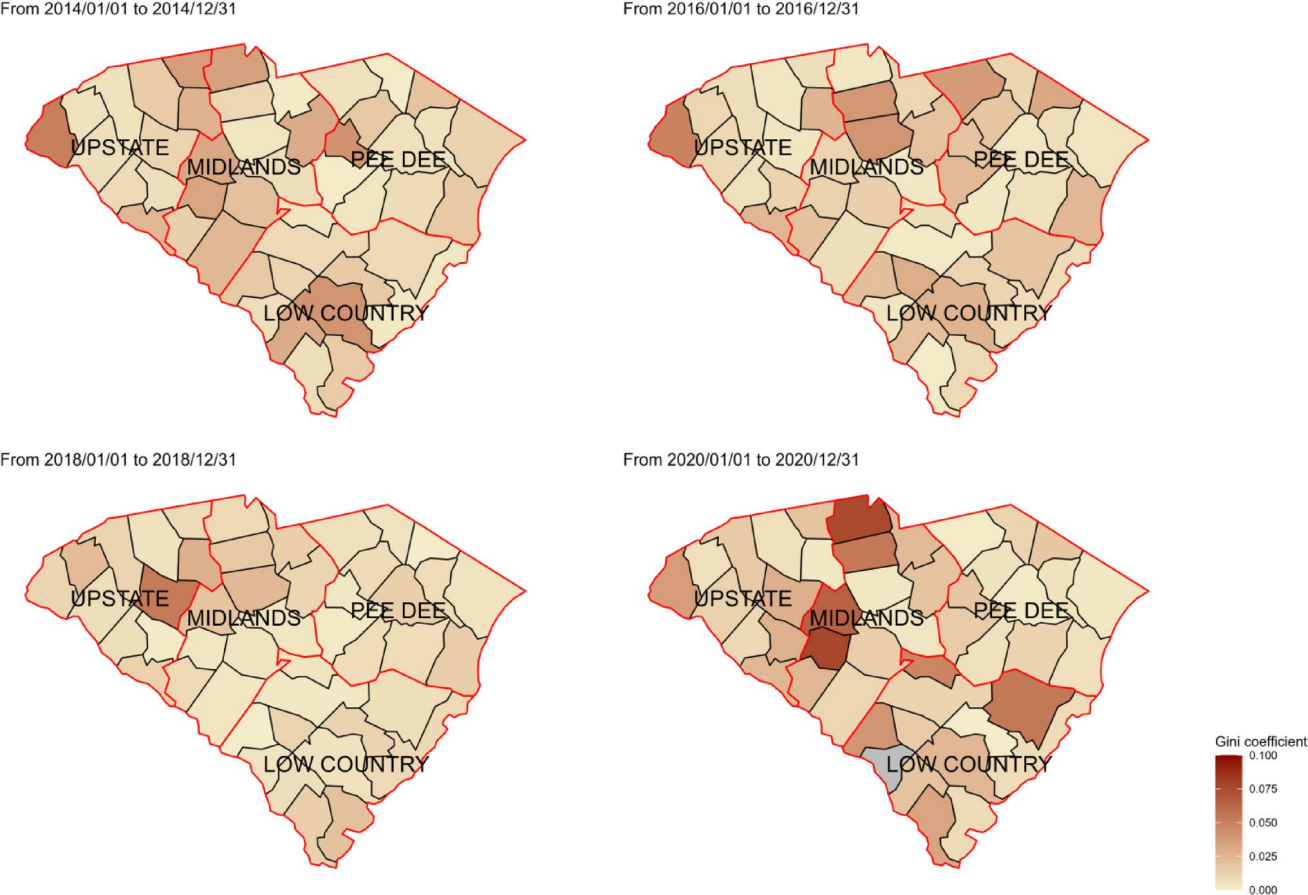
Geographic mapping for the Gini coefficient in South Carolina in 2014, 2016, 2018, and 2020

Additionally, the BWR fluctuated from 2013 to 2020 and was less than 1 in some years and greater than 1 in others, indicating that the RIC percentage among Black individuals was not consistently lower than that of White individuals throughout the study period. (Fig. 2) Geographically, counties in the southern and middle regions of SC exhibited consistently higher racial disparities than others when using ID to measure racial disparities in RIC (Fig. 3). However, counties in the northwest region of SC showed higher disparity when using Gini as the measurement (Fig. 4).

### The Distribution of County-Level Characteristics

Table 1 presents the distribution of county-level characteristics across 46 counties in SC in 2020. The median percentage of the male population and vacant homes were 48.43% and 17.08%, respectively. The unemployment rate stood at a median of 6.33%, while 10.38% of residents were uninsured. Additionally, 53.5% of the population adhered to a religious faith. On average, each county had just 1 Ryan White HIV center, 29 primary care providers, and three mental health facilities.

**Table 1:** Descriptive statistics for county-level variables^a^

	25th percentile	Median	75th percentile	IQR
Racial residential segregation				
Black/White dissimilarity index	25.00	30.50	38.00	13.00
Isolation index	0.41	0.54	0.61	0.19
Delta	0.31	0.46	0.56	0.24
Spatial proximity	0.93	0.95	0.97	0.04
Social capital indices				
Family unity	-1.81	-1.16	-0.51	1.30
Community health	-0.79	-0.55	-0.31	0.48
Institution health	-0.05	0.25	0.38	0.43
Collective efficacy	122.00	216.50	353.00	231.00
Socio vulnerability indices (SVI)				
SVI_Socioeconomic status	0.60	0.83	0.89	0.29
SVI_Household characteristics and disability	0.42	0.68	0.84	0.42
SVI_Minority status and language	0.60	0.71	0.79	0.19
SVI_Housing type and transportation	0.50	0.73	0.92	0.42
Health Care Resources and health behavior				
Primary care providers	8.00	29.00	74.00	66.00
Ryan White HIV centers	1.00	1.00	3.00	2.00
Mental health centers	3.00	3.00	4.00	1.00
Smoking (%)	17.00	19.00	20.00	3.00
Drinking (%)	15.00	16.00	17.00	2.00
Disability %	13.97	16.20	18.31	4.34
Other characteristics				
Male (%)	48.10	48.43	49.46	1.36
Vacant houses (%)	12.04	17.08	22.18	10.14
Unemployed (%)	5.03	6.33	7.31	2.28
Uninsured %	9.71	10.38	11.49	1.78
Gini index	0.45	0.47	0.48	0.03
Religious adherence (%)	41.00	53.50	59.00	18.00

IQR: Interquartile range

^a^25th percentile, median, 75th percentile, and IQR were only shown for county-level variables in year 2020, considering limited table space

### The Relationship between County-Level Characteristics and Racial Disparities in RIC

When using ID as the outcome, counties with exacerbated racial disparities in RIC were more likely to have less social interaction among Black individuals (indicated by isolation index; β=-1.92, 95%CI: -3.31~-0.53), stronger family unity (β = 1.75, 95%CI: 0.40 ~ 3.13), less collective efficacy (β=-1.27, 95%CI: -2.04~-0.52), fewer primary care providers (β=-1.78, 95%CI:-2.75~-0.81), and fewer income inequalities (indicated by Gini index; β=-0.81, 95%CI: -1.5~-0.08). For weighted ID, no county-level contextual factors explored were significantly associated with racial disparities in RIC. Similar to ID, when using the Gini coefficient as the outcome, counties with fewer primary care providers (β=-0.004, 95%CI: -0.006~-0.001) and less social capital in collective efficacy (β=-0.002, 95%CI: -0.005 ~ 0.000) showed higher degrees of racial disparities in RIC (Table 2).

**Table 2:** Association of county-level characteristics with Racial disparities in retention in care across 46 counties in South Carolina from 2013 to 2020

	Index of Disparity		Weighted Index of Disparity		Gini coefficient	
	Beta (95%CI)	p-value	Beta (95% CI)	p-value	Beta (95% CI)	p-value
Racial residential segregation						
Black/White dissimilarity index	0.37(-0.5, 1.23)	0.431	0(-0.5, 0.49)	0.997	0(-0.003, 0.002)	0.979
Isolation index	-1.92(-3.31, -0.53)	0.015	-0.01(-0.82, 0.8)	0.985	-0.0001(-0.004, 0.004)	0.952
Delta	0.17(-0.96, 1.32)	0.778	0.05(-0.61, 0.7)	0.899	0.0002(-0.003, 0.003)	0.922
Spatial proximity	0.56(-0.14, 1.25)	0.137	0.12(-0.29, 0.52)	0.583	0.0006(-0.001, 0.003)	0.546
Social capital indices						
Family unity	1.75(0.4, 3.13)	0.023	0.68(-0.09, 1.46)	0.121	0.0034(0, 0.007)	0.115
Community health	0.45(-0.21, 1.09)	0.215	-0.18(-0.56, 0.19)	0.386	-0.0009(-0.003, 0.001)	0.361
Institution health	0.92(0.19, 1.65)	0.024	0.21(-0.24, 0.65)	0.396	0.001(-0.001, 0.003)	0.395
Collective efficacy	-1.27(-2.04, -0.52)	0.004	-0.46(-0.95, 0.05)	0.102	-0.0024(-0.005, 0)	0.047
Socio vulnerability indices (SVI)						
SVI_Socioeconomic status	---		0.09(-0.81, 1.07)	0.867	---	
SVI_Household characteristics and disability	0.13(-0.93, 1.22)	0.822	-0.11(-0.71, 0.53)	0.75	-0.0005(-0.003, 0.003)	0.775
SVI_Minority status and language	0.58(-0.2, 1.36)	0.175	0.12(-0.34, 0.55)	0.635	0.0006(-0.002, 0.003)	0.61
SVI_Housing type and transportation	-0.23(-0.72, 0.27)	0.386	-0.26(-0.54, 0.02)	0.096	-0.0013(-0.003, 0)	0.094
Health Care Resources and health behavior						
primary care providers	-1.78(-2.75, -0.81)	0.002	-0.7(-1.34, -0.01)	0.065	-0.0037(-0.006, -0.001)	0.02
Ryan White HIV centers	0.31(-0.46, 1.1)	0.465	-0.03(-0.47, 0.42)	0.897	-0.0002(-0.002, 0.002)	0.89
Mental health centers	0.09(-0.68, 0.86)	0.836	-0.33(-0.79, 0.13)	0.204	-0.0016(-0.004, 0.001)	0.2
Smoking (%)	-0.13(-0.82, 0.56)	0.714	0.01(-0.41, 0.41)	0.972	0.0001(-0.002, 0.002)	0.929
Drinking (%)	0.18(-0.59, 0.94)	0.666	0.06(-0.39, 0.5)	0.805	0.0002(-0.002, 0.002)	0.83
Disability %	1.22(0.11, 2.32)	0.042	0.22(-0.44, 0.81)	0.526	0.0011(-0.002, 0.004)	0.53
Other characteristics						
Male (%)	0.58(-0.28, 1.43)	0.216	0.31(-0.17, 0.8)	0.254	0.0015(-0.001, 0.004)	0.255
Vacant houses (%)	0.26(-0.44, 0.99)	0.503	0.08(-0.3, 0.51)	0.71	0.0004(-0.002, 0.003)	0.713
Unemployed (%)	-0.82(-1.79, 0.14)	0.116	-0.1(-0.66, 0.42)	0.724	-0.0005(-0.003, 0.002)	0.722
Uninsured %	0.46(-0.45, 1.33)	0.337	-0.22(-0.74, 0.27)	0.423	-0.0011(-0.004, 0.001)	0.424
Gini index	-0.81(-1.5, -0.08)	0.033	-0.1(-0.5, 0.32)	0.643	-0.0005(-0.002, 0.002)	0.663
Religious adherence (%)	-0.16(-1.01, 0.67)	0.727	-0.06(-0.56, 0.42)	0.838	-0.0002(-0.003, 0.002)	0.865
Region						
MIDLANDS	-0.36(-2.14, 1.42)	0.709	0.67(-0.4, 1.72)	0.267	0.0035(-0.002, 0.009)	0.221
PEE DEE	-0.36(-1.91, 1.18)	0.671	-0.41(-1.38, 0.52)	0.443	-0.0019(-0.006, 0.002)	0.442
UPSTATE	2.46(-0.38, 5.31)	0.119	1.29(-0.42, 2.96)	0.177	0.0067(-0.001, 0.015)	0.139

Notes: --- Variables were omitted after forward selection

## Discussion

The current study investigated the spatiotemporal variations of four quantified measurements of racial disparities in RIC from 2013 to 2020 in SC and identified potential predictors contributing to these variations. Results from our study revealed no significant linear increase or decrease in racial disparities in RIC from 2013 to 2020, but there was clear spatial heterogeneity across counties in SC. When using ID and Gini to measure racial disparities, counties with more primary care providers and higher social capital in collective efficacy showed lower racial disparities in RIC. Regarding racial residential segregation, higher racial disparities in RIC were observed more in counties with a lower isolation index. Efforts to address racial disparities should continue, and innovative approaches, specifically those that focus on social and structural factors, should be developed and implemented for populations that have poor HIV outcomes in the US.

### Consistency and Inconsistency between the Four Measurements of Racial Disparities

Although consistent, insignificant increasing or decreasing trends were found for all four summary measurements of racial disparities in the present study. These measurements sometimes indicated different relationships between county-level characteristics and racial disparities. For example, the negative association between the number of healthcare providers and racial disparities was found for ID and the Gini coefficient, but not for the weighted ID. The relationship between racial residential segregation and racial disparities was observed for ID only, but not for the weighted ID and the Gini coefficient. The potential for inconsistencies between disparity measures due to the way they are calculated has been one of the well-known methodological issues for racial disparity measures [22]. However, experts in quantifying disparities have typically avoided promoting a single disparity measure as the “best” measure, and the use of multiple measures rather than one single measure was recommended [24, 27]. Following this guidance, our study employed four of the most commonly used summary measures of racial disparities to illustrate the relationship between county-level characteristics and racial disparities in RIC. This approach provided us with a more comprehensive understanding of the examined relationship.

### Factors Associated with Lower Racial Disparities in RIC

Our findings indicate that counties with more healthcare providers and higher collective efficacy demonstrated lower racial disparities in RIC. The number of primary care providers in a county reflects one aspect of local healthcare access, while the collective efficacy index measures one aspect of social capital, such as the community’s ability to work together to achieve common goals, mutual trust, and the willingness to take collective action for the common good [28, 29]. Primary care providers are key sources of HIV information for Black/African American communities [30], and people of color who rely on their primary healthcare providers as the main source of HIV care and information were more motivated to engage in HIV treatment [31]. Thus, a dwindling HIV workforce and a lack of coordinated HIV care exacerbated the problem of poor RIC, especially for Black/African American PWH [29]. As for collective efficacy or social capital in general, PWH in counties with high social capital can access more social interactions and resources, which have been proven to affect health-seeking behaviors and retention in medical appointments positively [12, 32].

### Racial Residential Segregation and Racial Disparities in RIC

Counties with a smaller isolation index, which indicates less racial residential segregation, exhibited greater racial disparities in RIC [33]. The isolation index focuses on the likelihood that a member of a particular racial group lives in a neighborhood with a high proportion of people from the same group [34]. Living in a neighborhood with a higher proportion of people from the same group can be related to stronger social networks and support systems, providing culturally appropriate health resources, enhancing health literacy, and improving health outcomes [35, 36]. Healthcare infrastructure and community support are crucial in reducing county-level racial disparities in RIC in SC [37–39]. Expanding the availability of HIV care services and promoting culturally appropriate community social support can increase treatment opportunities, ultimately helping to decrease racial disparities.

### Study Limitations

Our study has some limitations. First, we could not conduct more granular spatial analyses (i.e., ZIP code level instead of county level) due to a lack of geographic identification information at the ZIP code level in eHARs, which is necessary to link publicly available ZIP code-level characteristics. However, the variations of community factors and racial disparities in RIC within each county should not be ignored. Second, due to the threat of potential modifiable areal unit problems (MAUP) [40], caution must be taken when generating the findings to other geographic levels. Third, there was little data on institutional-level practices and characteristics (e.g., average wait time, provider demographics) that may influence racial disparities in RIC. Future studies should aim to include these institutional variables to provide a more comprehensive understanding of how structural factors influence unequal outcomes and can inform targeted interventions to reduce racial disparities. Lastly, this study focused solely on racial disparities between Black and White PWH due to the limited number of individuals from other racial groups in our dataset. Future research should incorporate additional racial and ethnic groups to provide a more comprehensive understanding of disparities in RIC.

## Conclusions

Despite efforts to curtail the HIV epidemic in the past decades, racial disparities in HIV care-related outcomes, such as RIC, remained in SC over the years. Interventions leveraging contextual and structural factors can be better designed to support equitable healthcare access and outcomes among PWH across racial and geographic populations. These interventions could include strengthening healthcare infrastructure in underserved areas and promoting community-level support systems. In addition, partnering with community leaders, advocacy groups, and PWH themselves will enhance the relevance and sustainability of these interventions, ultimately leading to better RIC and improved health outcomes across all racial and ethnic groups in SC.

## Electronic Supplementary Material

Below is the link to the electronic supplementary material.

Supplementary Material 1

## Data Availability

The authors are prohibited from making individual-level data available publicly due to provisions in our data use agreements with state agencies/data providers, institutional policy, and ethical requirements. To facilitate research, we make access to such data available via approved data access requests through the data owners. The data is unavailable externally or for re-release due to prohibitions in data use agreements with our state agencies or other data providers. Institutional policies stipulate that all external requests for data access require collaboration with an (author’s affiliation) researcher. For more information or to make a request, please contact (Bankole Olatosi, PhD): Olatosi@mailbox.sc.edu. The underlying analytical codes are available from the authors on request. Declarations. The authors declare that the research was conducted in the absence of any commercial or financial relationships that could be construed as a potential conflict of interest. The study protocol was approved by the Institutional Review Board at both the University of South Carolina and the South Carolina Department of Health and Environment Control. Not applicable. Not applicable.

## References

[1] Stricker SM, et al. Retention in care and adherence to ART are critical elements of HIV care interventions. AIDS Behav. 2014;18:465–75. 10.1007/s10461-013-0598-6 24292251

[2] Yehia BR, et al. Retention in care is more strongly associated with viral suppression in HIV-infected patients with lower versus higher CD4 counts. JAIDS J Acquir Immune Defic Syndr. 2014;65(3):333–9. 24129370 10.1097/QAI.0000000000000023 PMC3945404

[3] Giordano TP, et al. Factors associated with the use of highly active antiretroviral therapy in patients newly entering care in an urban clinic. JAIDS J Acquir Immune Defic Syndr. 2003;32(4):399–405. 12640198 10.1097/00126334-200304010-00009

[4] Organization WH. The role of HIV viral suppression in improving individual health and reducing transmission: policy brief. World Health Organization; 2023.

[5] The center for disease control. and prevention, *Estimated HIV incidence and prevalence in the UnitedStates, 2018–2022*. https://link.springer.com/journal/10461/submission-guidelines. Accessed 20 May 2024.

[6] Maheu-Giroux M, Mishra S. Evidence with 95-95-95 that ambitious is feasible. Lancet HIV. 2024;11(4):e203–4. 38467134 10.1016/S2352-3018(24)00028-6

[7] Sheehan DM, et al. Retention in HIV care and viral suppression: individual-and neighborhood-level predictors of racial/ethnic differences, florida, 2015. AIDS Patient Care STDs. 2017;31(4):167–75. 28414260 10.1089/apc.2016.0197 PMC5397217

[8] Anderson AN, et al. Disparities in retention in care among adults living with HIV/AIDS: a systematic review. AIDS Behav. 2020;24:985–97. 31555931 10.1007/s10461-019-02679-2

[9] Rebeiro PF, et al. Geographic variations in retention in care among HIV-infected adults in the united States. PLoS ONE. 2016;11(1):e0146119. 26752637 10.1371/journal.pone.0146119 PMC4708981

[10] Bulsara SM, Wainberg ML, Newton-John TR. Predictors of adult retention in HIV care: a systematic review. AIDS Behav. 2018;22:752–64. 27990582 10.1007/s10461-016-1644-y PMC5476508

[11] Eberhart MG, et al. Individual and community factors associated with geographic clusters of poor HIV care retention and poor viral suppression. JAIDS J Acquir Immune Defic Syndr. 2015;69:S37–43. 25867777 10.1097/QAI.0000000000000587 PMC4568746

[12] Zeng C, et al. County-level predictors of retention in care status among people living with HIV in South Carolina from 2010 to 2016: a data-driven approach. AIDS. 2021;35(Suppl 1):pS53. 10.1097/QAD.0000000000002832 PMC8098716 33867489

[13] Filippone P, et al. Understanding why racial/ethnic inequities along the HIV care continuum persist in the united states: a qualitative exploration of systemic barriers from the perspectives of African american/black and Latino persons living with HIV. Int J Equity Health. 2023;22(1):168. 37649049 10.1186/s12939-023-01992-6 PMC10466874

[14] Doshi RK, Bowleg L, Blankenship KM. Tying structural racism to human immunodeficiency virus viral suppression. Oxford University Press US; 2021. pp. e646–8. 10.1093/cid/ciaa1252 32845976

[15] Friedman SR, et al. Toward a theory of the underpinnings and vulnerabilities of structural racism: looking upstream from disease inequities among people who use drugs. Int J Environ Res Public Health. 2022;19(12):7453. 35742699 10.3390/ijerph19127453 PMC9224240

[16] McCree DH, et al. Exploring changes in racial/ethnic disparities of HIV diagnosis rates under the ending the HIV epidemic: A plan for America initiative. Public Health Rep. 2020;135(5):685–90. 32762633 10.1177/0033354920943526 PMC7485057

[17] *Centers for Disease Control and Prevention. Sexually Transmitted Disease Surveillance, 2011. Atlanta: U.S. Department of Health and Human Services;* 2012. https://www.cdc.gov/std/stats/archive/Surv2011.pdf. *Accessed 15 Jun 2024.*

[18] Bilal U, et al. Racial/ethnic and neighbourhood social vulnerability disparities in COVID-19 testing positivity, hospitalization, and in-hospital mortality in a large hospital system in pennsylvania: A prospective study of electronic health records. Lancet Reg Health-Americas. 2022;10:100220. 10.1016/j.lana.2022.100220 PMC8891851 35262038

[19] Hall HI, Byers RH, Ling Q, Espinoza L. Racial/ethnic and age disparities in HIV prevalence and disease progression among men who have sex with men in the united States. Am J Public Health. 2007;97(6):1060–6. 17463370 10.2105/AJPH.2006.087551 PMC1874211

[20] Khazanchi R, et al. Neighborhood deprivation and racial/ethnic disparities in human immunodeficiency virus viral suppression: a single-center, cross-sectional study in the united States Midwest. Clin Infect Dis. 2021;72(10):e642–5. 32845985 10.1093/cid/ciaa1254

[21] Hoover K, Bohm M, Keppel K. Measuring disparities in the incidence of sexually transmitted diseases. Sex Transm Dis. 2008;35(12):S40–4. 18836391 10.1097/OLQ.0b013e3181886750

[22] Chesson HW, et al. Using reported rates of sexually transmitted diseases to illustrate potential methodological issues in the measurement of Racial and ethnic disparities. Sex Transm Dis. 2017;44(9):513–8. 28809767 10.1097/OLQ.0000000000000646 PMC6727205

[23] Yang X, et al. Patterns and predictors of racial/ethnic disparities in HIV care continuum in the Southern USA: protocol for a population-based cohort study. BMJ Open. 2023;13(12):e080521. 38086599 10.1136/bmjopen-2023-080521 PMC10729084

[24] Hogg RS. Understanding the HIV care continuum. Lancet HIV. 2018;5(6):e269–70. 29893238 10.1016/S2352-3018(18)30102-4

[25] Chesson HW, Patel CG, Gift TL, Aral SO. Trends in selected measures of Racial and ethnic disparities in gonorrhea and syphilis in the united states, 1981–2013. Sex Transm Dis. 2016;43(11):661–7. 27893593 10.1097/OLQ.0000000000000518 PMC5905678

[26] Menza TW, Hixson LK, Lipira L, Drach L. *Social determinants of health and care outcomes among people with HIV in the United States.* Open Forum Infectious Diseases, 2021. Vol. 8. No. 7. US: Oxford University Press. 10.1093/ofid/ofab330 PMC8297699 34307729

[27] Rossen LM, Schoendorf KC. Measuring health disparities: trends in racial– ethnic and socioeconomic disparities in obesity among 2-to 18-year old youth in the united states, 2001–2010. Ann Epidemiol. 2012;22(10):698–704. 22884768 10.1016/j.annepidem.2012.07.005 PMC4669572

[28] Lee M. *US Congress Joint Economic Committee The geography of social capital in America*. 2018. https://www.lee.senate.gov/services/files/da64fdb7-3b2e-40d4-b9e3-07001b81ec31. Accessed 15 Jun, 2024.

[29] Byrd KK, et al. Retention in HIV care among participants in the patient-centered HIV care model: a collaboration between community-based pharmacists and primary medical providers. AIDS Patient Care STDs. 2019;33(2):58–66. 30648888 10.1089/apc.2018.0216 PMC6379900

[30] Carter G, et al. Primary care providers as a critical access point to HIV information and services for African American and Latinx communities. PLoS ONE. 2021;16(2):e0246016. 33539465 10.1371/journal.pone.0246016 PMC7861398

[31] Levison JH, et al. Where it falls apart: barriers to retention in HIV care in Latino immigrants and migrants. AIDS Patient Care STDs. 2017;31(9):394–405. 28891715 10.1089/apc.2017.0084 PMC5610398

[32] Browning CR, Burrington LA, Leventhal T, Brooks-Gunn J. Neighborhood structural inequality, collective efficacy, and sexual risk behavior among urban youth. J Health Soc Behav. 2008;49(3):269–85. 18771063 10.1177/002214650804900303 PMC3111971

[33] Massey DS, Denton NA. The dimensions of residential segregation. Soc Forces. 1988;67(2):281–315.

[34] White K, Borrell LN. Racial/ethnic residential segregation: framing the context of health risk and health disparities. Health Place. 2011;17(2):438–48. 21236721 10.1016/j.healthplace.2010.12.002 PMC3056936

[35] Sherwood J, Blumenthal S, Sayas A. *White Counties Stand Apart: The Primacy of Residential Segregation in COVID-19 and HIV Diagnoses.* 10.1089/apc.2020.0155 PMC7585613 32833494

[36] Williams DR, Collins C. *Racial residential segregation: a fundamental cause of racial disparities in health.* Public health reports, 2016. 10.1093/phr/116.5.404 PMC1497358 12042604

[37] Ransome Y, Kawachi I, Dean LT. Neighborhood social capital in relation to late HIV diagnosis, linkage to HIV care, and HIV care engagement. AIDS Behav. 2017;21(3):891–904. 27752875 10.1007/s10461-016-1581-9 PMC5306234

[38] Ransome Y, et al. How do social capital and HIV/AIDS outcomes geographically cluster and which sociocontextual mechanisms predict differences across clusters? J Acquir Immune Defic Syndr. 2017;76(1):13. 28797017 10.1097/QAI.0000000000001463 PMC5584611

[39] Mukoswa GM, Charalambous S, Nelson G. The association between social capital and HIV treatment outcomes in South Africa. PLoS ONE. 2017;12(11):e0184140. 29121656 10.1371/journal.pone.0184140 PMC5679596

[40] Jelinski DE, Wu J. The modifiable areal unit problem and implications for landscape ecology. Landscape Ecol. 1996;11:129–40.

