# Supplementary material for: Longitudinal Patterns and Predictors of Racial Disparities of HIV Retention in Care: A Statewide Cohort Analysis
Source: AIDS Behav. 2025 Jul 16;29(12):3746–56. doi: 10.1007/s10461-025-04813-9 (PMC12580437; doi:10.1007/s10461-025-04813-9)
Supplement: Supplementary file 1 — Supplementary Material 1 [file 10461_2025_4813_MOESM1_ESM.docx]

| **Supplemental Table 1. The detailed definition for each county-level variable** | |
| --- | --- |
| Variables | Definitions |
| **Racial residential segregation** |  |
| Black/White dissimilarity index | The percentage of either Black or White residents that would have to move to different geographic areas to produce a distribution that matches that of the larger area |
| Isolation index | The probability that the Black group member would encounter another Black group member |
| Delta | The relative amount of physical space occupied by the Black group |
| Spatial proximity | The extent to which neighborhoods inhabited by Black members adjoin one another |
| **Social capital indices** |  |
| Family unity | The share of births that are to unwed mothers, children living in single-parent families, and women aged 35-44 who are married |
| Community health | Non-religious non-profits per capita, congregations per capita, and the informal civil society subindex |
| Institution health | Presidential voting rate, census response rate, and confidence subindex |
| Collective efficiency | Violent crimes per 100,000 people |
| **Socio vulnerability indices (SVI)** |  |
| SVI_Socioeconomic status | Below 150% poverty, unemployed, housing cost burden, no high school diploma, no health insurance |
| SVI_Household characteristics and disability | Aged 65 or older, aged 17 or younger, civilian with a disability, single-parent households, English language proficiency |
| SVI_Minority status and language | Hispanic or Latino (of any race); Black and African American, Not Hispanic or Latino; American Indian and Alaska Native, Not Hispanic or Latino; Asian, Not Hispanic or Latino; Native Hawaiian and Other Pacific Islander, Not Hispanic or Latino; Two or More Races, Not Hispanic or Latino; Other Races, Not Hispanic or Latino |
| SVI_Housing type and transportation | Multi-unit structures, mobile homes, crowding, no vehicle, group quarters |
| **Health Care Resources and health behavior** |  |
| primary care providers | Number of primary care providers per 100,000 population |
| Ryan White HIV centers | Number of Ryan White HIV centers per newly diagnosed HIV cases each year within 25 miles radius |
| Mental health centers | Number of mental health centers per newly diagnosed HIV cases each year within 25 miles radius |
| Smoking (%) | Percent of adults who are current smokers |
| Drinking (%) | Percent of adults reporting binge or heavy drinking |
| Disability % | Percentage of adults with a disability (between ages 18 and 64) |
| **Other characteristics** |  |
| Male (%) | Percent of male persons |
| Vacant houses (%) | Percentage of housing units vacant |
| Unemployed (%) | Percent of 16 years and older persons who are unemployed |
| Uninsured % | Percent of occupied housing units without access to a vehicle |
| Gini index | Income inequality represented by a statistical measure of income dispersion |
| Religious adherence (%) | Percent of persons with religious adherence |

| **Supplemental Table 2. Demographic characteristics of all PWH included in the analyses from 2013 to 2020** | |
| --- | --- |
|  | **People with HIV, n (%)** |
| **Total** | 17,591 (100.00%)^a^ |
| **Age group** |  |
| 18-30 | 6,021 (34.2%) |
| 30-40 | 5,084 (28.9%) |
| 40-50 | 3,983 (22.6%) |
| 50-60 | 1,900 (10.8%) |
| >60 | 603 (3.4%) |
| **Sex/gender** |  |
| Female | 4,896 (27.8%) |
| Male | 12,695 (72.2%) |
| **Race** |  |
| Non-Hispanic Black | 13,360 (75.9%) |
| Non-Hispanic White | 4,231 (24.1%) |
| **HIV transmission model** |  |
| Heterosexual | 4,350 (24.7%) |
| Injection drug use | 1,599 (9.1%) |
| Men who have sex with men | 7,949 (45.2%) |
| Others | 3,693 (21.0%) |
| Notes: ^a^The number 17,591 reflects the total number of individuals over time, including those who may have dropped out in certain years. The cohort size may vary each year due to new patient enrollments and attrition. | |
